# Analysis of Human Gut Microbiota Composition Associated to the Presence of Commensal and Pathogen Microorganisms in Côte d’Ivoire

**DOI:** 10.3390/microorganisms9081763

**Published:** 2021-08-18

**Authors:** Veronica Di Cristanziano, Fedja Farowski, Federica Berrilli, Maristella Santoro, David Di Cave, Christophe Glé, Martin Daeumer, Alexander Thielen, Maike Wirtz, Rolf Kaiser, Kirsten Alexandra Eberhardt, Maria J. G. T. Vehreschild, Rossella D’Alfonso

**Affiliations:** 1 Institute of Virology, Faculty of Medicine and University Hospital of Cologne, University of Cologne, 50935 Cologne, Germany; maike.wirtz@uk-koeln.de (M.W.); rolf.kaiser@uk-koeln.de (R.K.); 2 Department I of Internal Medicine, Faculty of Medicine and University Hospital of Cologne, University of Cologne, 50937 Cologne, Germany; fedja.farowski@uk-koeln.de (F.F.); maria.vehreschild@uk-koeln.de (M.J.G.T.V.); 3 Department of Internal Medicine, Infectious Diseases, University Hospital Frankfurt, Goethe University Frankfurt, 60590 Frankfurt am Main, Germany; 4 Department of Clinical Sciences and Translational Medicine, University of Rome Tor Vergata, 00133 Rome, Italy; berrilli@uniroma2.it (F.B.); maristella5384@gmail.com (M.S.); dicave@uniroma2.it (D.D.C.); 5 Centre Don Orione Pour Handicapés Physiques, Bonoua BP 21, Côte d’Ivoire; possombo@gmail.com (C.G.); dalfonso@uniroma2.it (R.D.); 6 Seq-IT GmbH & Co KG, 67655 Kaiserslautern, Germany; m.daeumer@immungenetik-kl.de (M.D.); a.thielen@immungenetik-kl.de (A.T.); 7 Department of Tropical Medicine, Bernhard Nocht Institute for Tropical Medicine & I. Department of Medicine, University Medical Center Hamburg-Eppendorf, 20359 Hamburg, Germany; k.eberhardt@bnitm.de; 8 Institute for Transfusion Medicine, University Medical Center Hamburg-Eppendorf, 20251 Hamburg, Germany; 9 German Center for Infection Research (DZIF), Partner Site Bonn, 50937 Cologne, Germany; 10 Department of Systems Medicine, University of Rome Tor Vergata, 00133 Rome, Italy

**Keywords:** intestinal co-infection, biotype, virus, bacteria, *G. duodenalis*, *Blastocystis*, *Entamoeba coli*, *Entamoeba dispar*, *Entamoeba hartmanni*

## Abstract

Background: The human gut microbiota is a microbial ecosystem contributing to the maintenance of host health with functions related to immune and metabolic aspects. Relations between microbiota and enteric pathogens in sub-Saharan Africa are scarcely investigated. The present study explored gut microbiota composition associated to the presence of common enteric pathogens and commensal microorganisms, e.g., *Blastocystis* and *Entamoeba* species, in children and adults from semi-urban and non-urban localities in Côte d’Ivoire. Methods: Seventy-six stool samples were analyzed for microbiota composition by 16S rRDNA sequencing. The presence of adeno-, entero-, parechoviruses, bacterial and protozoal pathogens, *Blastocystis*, and commensal *Entamoeba* species, was analyzed by different molecular assays. Results: Twelve individuals resulted negative for any tested microorganisms, 64 subjects were positive for one or more microorganisms. Adenovirus, enterovirus, enterotoxigenic *Escherichia coli* (ETEC), and *Blastocystis* were frequently detected. Conclusions: The bacterial composition driven by Prevotellaceae and Ruminococcaceae confirmed the biotype related to the traditional dietary and cooking practices in low-income countries. Clear separation in UniFrac distance in subjects co-harboring *Entamoeba hartmanni* and *Blastocystis* was evidenced. Alpha diversity variation in negative control group versus only *Blastocystis* positive suggested its possible regulatory contribution on intestinal microbiota. Pathogenic bacteria and virus did not affect the positive outcome of co-harbored *Blastocystis*.

## 1. Introduction

The intestinal microbiota consists of a complex microbial community, which exhibits a mutualistic relationship with the host and a crucial impact on human health [1]. About 60% of its bacterial composition persists stably for decades (e.g., the phyla Firmicutes, Bacteroidetes and Actinobacteria), while the remaining proportion is involved in transient modifications induced by different environmental conditions.

The high diversity and the relative abundance of beneficial bacterial strains, compared to those potentially harmful, promote the homeostasis of intestinal functions and host-protective immunity [2], and the combinations of redundant microbial species, differing among the human populations and geographical areas, insure fundamental metabolic functions [3]. However, several factors, e.g., antibiotic therapies, may lead to the loss of microbial diversity and imbalances between commensal versus pathogenic bacterial strains, weakening the recovery of intestinal microbiota [4].

The development of advanced sequencing techniques has allowed a more comprehensive understanding of the relationships between intestinal bacteria and the human host than a culture-based analysis alone [1]. Increasing evidence suggests a distinction between a state of balanced homeostasis, typical of healthy subjects, and different dynamic associations of microbial communities that seem to predispose to non-communicable diseases [5], as supported by studies on animal models [6]. 

Pioneering microbiota studies were performed in Europe, China, and the United States, evidencing a lower microbial diversity compared to pre-industrial populations [7,8]. Further comparisons have been carried out between different ethnic groups living in America [9,10] and a growing number of data relating to African populations are now available [11,12,13,14].

The characterization of human intestinal microbiota, especially in endemic environments for enteropathy, cannot neglect the potential presence of common intestinal pathogens, including bacteria, viruses, and parasites, and their influence on microbiota balance [15,16]. However, the dynamics of relationships between pathogens and commensals, both prokaryotes and eukaryotes, are often underestimated [17,18] or focused on single microorganisms [19,20,21]. Poor water, sanitation, and hygiene conditions in low and middle-income countries sustain the susceptibility to multiple and asymptomatic infections [22]. 

The link between natural systems, human health, and infections remains an interesting challenge. In line with these aspects, the present study investigated the gut microbiota in children and adults living in Côte d’Ivoire. A particular, focus was on the changes in microbiota composition related to the presence of *Blastocystis,* commensal *Entamoeba* species, and different common enteric pathogens.

## 2. Materials and Methods

### 2.1. Study Area and Sample Collection

Human fecal samples were collected at four sites in the department of Grand-Bassam, in the south of Côte d’Ivoire. Bonoua (5°16′17″ N, 3°35′40″ W) was the only semi-urban locality while Assouindé (5°09′57.1″ N 3°28′10.9″ W), Kimoukro, and Yaou (5°14′25.0″ N 3°37′37.9″ W) were rural/semi-rural villages 6 to 20 km away from Bonoua. Sampling was performed during three distinct seasons characterizing the study area: the heavy rainy season (HRS), the low rainy season (LRS), and the heavy dry season (HDS).

### 2.2. Sample Management and Fecal Nucleic Acids Extraction

From each patient, one fecal sample was collected using an appropriate sterile container. Recent or ongoing antibiotic therapy constituted an exclusion principle. After allocation of an anonymous code, two aliquots (ca. 400 mg each) were prepared from the sample. The first aliquot was preserved for parasitological and NGS analysis by adding 0.5 mL of Qiagen Allprotect Tissue Reagent (QIAGEN, Hilden, Germany) in a sterile Nalgene^®^ cryogenic vial size 2.0 mL, frozen at −20 °C until transported to the University of Rome Tor Vergata (Italy). DNA from this aliquot was extracted by QIAmp Stool Mini Kit (QIAGEN, Hilden, Germany) [16]. The second aliquot for the xTAG GPP assay analysis and real-time PCR was frozen at −20 °C, transported to the University of Rome Tor Vergata, preserved at −80 °C until shipment to the Institute of Virology of the University of Cologne (Germany). In this case, sample preparation was performed using the automated platform VERSANT kPCR Molecular System (Siemens Healthcare Diagnostics, Erlangen, Germany), according to the manufacturer’s instructions, as described in [23].

### 2.3. xTAG GPP Assay

Stool samples were investigated for 15 human enteric pathogens by the xTAG Gastrointestinal Pathogen Panel (xTAG GPP) (Luminex Molecular Diagnostics, Toronto, ON, Canada), as already described [23]. The assay concurrently identified adenovirus subtypes 40/41, norovirus genogroup I and II (GI/GII), group A rotavirus, *Campylobacter* spp., *Clostridium difficile* toxin A/B, *Escherichia coli* O157, enterotoxigenic *Escherichia coli* (ETEC) LT/ST, *Salmonella* spp., Shiga-like toxin producing *E. coli* (STEC) stx1/stx2, *Shigella* spp., *Vibrio cholerae*, *Yersinia enterocolitica*, *Cryptosporidium hominis*, *Cryptosporidium parvum*, *Entamoeba histolytica*, *Giardia duodenalis*.

### 2.4. Detection of Adeno-, Entero-, and Parechoviruses by Real-Time PCR

The molecular detection of enteroviruses (EV) and parechoviruses (PeV) was performed as already described [24]. Adenoviruses (AdV) were detected by RealStar^®^ Adenovirus PCR Kit 1.0 (Altona Diagnostics, Hamburg, Germany).

### 2.5. Blastocystis and Commensal Entamoeba spp. Detection and Identification

End-point PCR and sequence analysis of the SSU rDNA region were carried out for the molecular identification of *Blastocystis* and *Entamoeba* species. The detection of *Blastocystis* was performed by amplification of a 600 bp fragment by nested PCR using the primers RD5-BhRDr and Blasto2F-Blasto2R [25] as described in D’Alfonso et al. [26]. The amplification of the specific fragment of *Entamoeba* spp., including the pathogenic *E. histolytica*, was obtained using the primers JVC and DSPR2 [27]. The amplicons were from 622 to 667 bp long depending on the species.

All amplicons were purified using a QIAquick Gel Extraction Kit (QIAGEN, Valencia, CA, USA) and sequencing was performed in the Bio-Fab Research Laboratory in Rome (Italy). The identities of the obtained sequences were verified using the Basic Local Alignment Search Tool (BLAST).

### 2.6. 16S rRNA Gene Amplicon Sequencing

PCR reactions for amplification of the V3 and V4 regions of bacterial 16S rDNA genes contained 25 μL reaction volume per sample: 2.5 µL microbial genomic DNA (12.5 ng), 1 µL of each primer (10 µM): 16S Amplicon PCR Forward Primer

(5′-TCGTCGGCAGCGTCAGATGTGTATAAGAGACAGCCTACGGGNGGCWGCAG-3〲) and 16S Amplicon PCR Reverse Primer

(5′-GTCTCGTGGGCTCGGAGATGTGTATAAGAGACAGGACTACHVGGGTATCTAATCC-3〲), 4 µL HF buffer, 2 µL dNTPs (10 mM each; Axon lab, Germany), 0.4 µL BSA (20 mg/mL, ThermoFisher Scientific, Waltham, MA, USA), 4 µL Betaine (5M, Sigma-Aldrich), and 0.2 µL Phusion Hifi DNA polymerase (ThermoFisher Scientific, Waltham, MA, USA). Primers for the amplification of the 16S V3 and V4 region were selected from Klindworth et al. [28]. Illumina adapter overhang nucleotide sequences were added to the gene-specific sequences (Illumina P5/P7 adaptor sequences in italic, see above).

Amplification was performed as follows: initial denaturation at 95 °C for 5 min, 25 cycles of denaturation at 95 °C for 40 s, annealing at 53 °C for 40 s, and extension at 72 °C for 60 s, followed by a final extension of 7 min at 72 °C. Before the “library preparation” by index-PCR, amplicons were purified using the Agencourt AMPure^®^XP system on a BioMek NX workstation (Beckman Coulter, Germany) following instructions provided by the manufacturer. The index PCR attached dual indices using the Nextera XT^®^ (Illumina Inc., San Diego, CA, USA) Index Kit following instructions provided by the manufacturer. The index PCR reaction was purified as described above followed by a library quantification, normalization, pooling, library denaturing, and MiSeq^®^ (Illumina Inc., San Diego, CA, USA) sample loading in a final concentration of 10 pM, following the protocol described by the manufacturer. Sequencing was accomplished using MiSeq reagent kit v2 in a 2 × 250 cycle paired-end sequencing run.

### 2.7. 16S rRNA Amplicon Data Processing

Sequencing data were processed using the QIIME DADA2 plugin with the denoise-paired option and standard parameters (trunc_q = 2, max_ee = 2, chimera_method = consensus). Taxonomic classification was performed by a Naïve Bayes classifier (sklearn) [29], which was trained on the SILVA database release 128 [30]. Rarefaction curves were determined based on the feature table and analysis of the relative proportion of each bacterial taxon was made after the data were rarefied at a sequencing depth of 4000 sequences per sample.

Statistical analyzes were carried out using R for Statistical Computing (version 3.5.1, R Foundation for Statistical Computing, Vienna, Austria) [31] (Team RC). The QIIME biom data were imported and diversity scores calculated using the phyloseq R package [31,32]. Rarefaction curves were determined based on the feature table and analysis of the relative proportion of each bacterial taxon was made after the data were rarefied at a sequencing depth of 4000 sequences per sample. All continuous data were presented as mean and standard deviation (SD) or median and range, tested with Student’s t-test, Mann–Whitney U test, and Kruskal–Wallis-test with Dunn’s post-test, as appropriate. In the beta diversity, the UniFrac distances between the samples were visualized using principal coordinate analysis (PCoA), and group effects were tested by a permutational multivariate analysis of variance (PERMANOVA). Differentially abundant taxa were identified using linear discriminant analysis (LDA) effective size (LefSe) [33]. All statistical tests were two-tailed, and a *p*-value < 0.05 was considered statistically significant.

## 3. Results

### 3.1. Study Area and Host Population

Sixty-seven fecal samples were collected from subjects aged between 1 to 59 years old and nine samples from infants (<1 year), representing four different localities: 53 samples from an urban area (Bonoua) and 23 samples from non-urban areas (Assouindé, Kimoukro, Yaou; see Table 1). Fourteen samples were collected during the heavy rainy season, 22 during the low rainy season, and 40 during the heavy dry season.

### 3.2. Viral, Bacterial, and Protozoa Detection

Within the cohort, 12 subjects, including four infants, were tested negative for all analyzed microorganisms; 64 subjects, including five infants, were tested positive for at least one virus, bacterium, or protozoa; 10 subjects, including three infants, were tested positive for a single microorganism, 20 subjects for two microorganisms, and 34 subjects, including two infants, for three or more microorganisms.

Overall, 32 subjects, including five infants, were tested positive for at least one virus, with adenovirus (AdV) and enterovirus (EV) being the most common, as showed in Table 2.

A total of 17 subjects, including two infants, were tested positive for bacterial pathogens with enterotoxigenic *E. coli* (ETEC), *Campylobacter*, *Shigella*, and *E. coli* O157 being the most frequently identified (Table 2). A total of 54 subjects, including one infant, were positive for at least one protozoa species with *Blastocystis* being the most common (47 out of 54). Overall, 20 subjects were positive for *Entamoeba hartmanni*, 11 for *Entamoeba coli* (*En. coli*), and two for *Entamoeba dispar*; *Giardia duodenalis* was detected in 25 individuals, including one infant (Table 3).

### 3.3. Gut Microbiota Composition

#### 3.3.1. Age

**Figure 1:**
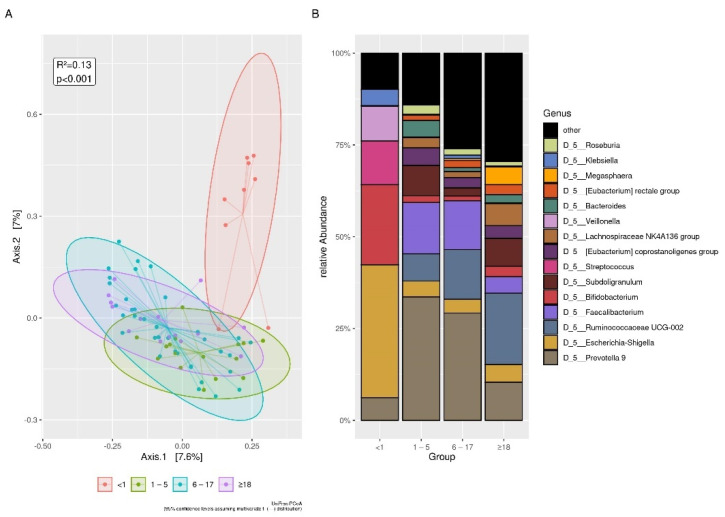
Differences between age groups. (**A**): UniFrac distances among infants and other age groups. The samples were visualized using principal coordinate analysis (PCoA). Differences with other age groups are statistically significant. (**B**): The plot of top 15 relative bacterial abundances at genus level. The statistical significances are shown in Table S1A.

A marked difference in diversity was found between infants compared to the other age groups (Figure 1A). Infants showed a significant lower alpha diversity, in particular when compared to those aged 6–17 and >18 years (Table 4).

As for bacterial composition, the phyla Proteobacteria and Actinobacteria were more abundant in <1 year of age, and the Firmicutes and Bacteroidetes phyla were more abundant in other age groups. In detail, the Christensenellaceae, Rikenellaceae, and Succinivibrionaceae families were absent in subjects <1 year of age, while a high prevalence of Actinomycetaceae was observed within this group (Figure S1A). Moreover, Enterobacteriaceae abundance (0.30025 (0.0005–1)) was higher when compared to subjects aged >1–5 (0.002875 (0.00025–0.26)), 6–17 (0.00375 (0–0.67675)), and ≥18 years (0.004 (0–0.32125)). An opposite trend was observed with Prevotellaceae (0 (0–0.5625)), which was less abundant in subjects <1 of age than in age groups 1–5 (0.3544 (0.00–0.8035), *p* < 0.05) and 6–17 years (0.2610 (0.00–0.9328), *p* < 0.001). Ruminococcaceae abundance (0.00025 (0–0.002)) was also lower than in the age groups 1–5 (0.29280 (0.06200–0.8785), *p* < 0.01), 6–17 years (0.33000 (0.0010–0.9120), *p* < 0.001), and >18 years (0.33000 (0.0010–0.9120), *p* < 0.001). Finally, *Eubacterium coprostanoligenes*, *Eubacterium rectale* group, *Lachnospiracee* NK group, *Roseburia*, *Ruminococcaceae* UCG-010, *Ruminococcaceae* UCG-013, *Christensenellaceae* R-7 group, and *Rikenellaceae* RC9 were absent in subjects aged <1 year.

The organisms most frequently present at the genus level are shown in Figure 1B; the related statistical significance in the abundance comparison between infants versus all other groups is reported in Table S1A.

#### 3.3.2. Denovo Clustering

In an attempt to identify different gut microbiome enterotypes in all 67 subjects >1 year of age, we calculated the UniFrac distances between all samples, partitioned (clustered) the data into k clusters around meteroids (Figure S2A), and calculated the respective gap statistics. Using this approach, we were able to split the subjects into two clusters. The first one, grouping 39 subjects aged 1–59 years and showed higher richness based on Chao1 (*p* < 0.01), OTUs (*p* < 0.01), and PD (*p* < 0.001), and dominance of Ruminococcaceae (Clostridia Class) over Prevotellaceae (Bacteroidia Class). The second cluster, including 28 subjects aged 1–19 years, showed an inverse dominance of Prevotellaceae versus Ruminococcaceae (Figure S2B–D). At the genus level, both clusters had similar abundances of *Bacteroides*, *Escherichia-Shigella*, *Faecalibacterium*, *Roseburia*, *Eubacterium*, *Lachnospiraceae*, *Christensenellaceae* R-7 group.

#### 3.3.3. Sex

**Figure 2:**
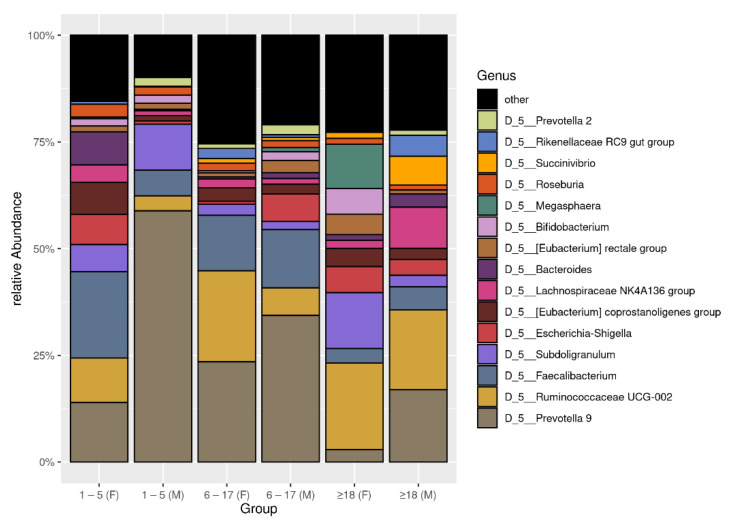
The plot of top 15 relative bacterial abundances at genus level. The subjects were distinguished by sex and age.

Overall, a significant difference in Faith’s phylogenetic diversity (PD) between 36 females (16.56 (0.9572–29.68)) and 40 males (12.22 (1.6780–34.15)) (*p* < 0.05) was observed. Ruminococcaceae abundance was higher in females (0.41125 (0–0.912)) versus males (0.174875 (0–0.8527)) (*p* < 0.01) and the *Ruminococcaceae* UCG-002 genus abundance showed the same trend (*p* < 0.01). No significant differences in alpha and beta diversity were found between male and female infants. Among subjects of other age groups, only females aged 6–17 years old showed a PD index between (19.28 (12.50–29.68)) which was different compared to males of 1–5 years old (12.39 (7.27–15.23)) (*p* < 0.05) and 6–17 years old (13.07 (7.34–34.15)) (*p* < 0.05). Among subjects aged 1–5 years, there was a lower abundance of *Prevotella* 9 in females (0.003 (0–0.477)) compared to males (0.704 [0.012–0.741]) (*p* < 0.01). In the 6–17 years age group, there was a greater abundance of *Ruminococcaceae* UCG-002 in females (0.125 (0.009–0.827)) compared to males (0.015 (0–0.440)) (*p* < 0.05). Within subjects >18 years of age, there was a higher abundance of *Megasphaera* in females (0.017 (0–0.654)) compared to males (0.00 (0–0)) (*p* < 0.01). Between the age groups, only *Prevotella* 9 was less abundant in females >18 (0.002 (0–0.139)) versus males of 1–5 years of age (0.704 (0.012–0.741) (*p* < 0.01). The top 15 bacterial composition at the genus level was shown in Figure 2.

Subsequently, the results relating to the nine infants are not indicated considering their small number and the unexpected variety of isolated pathogens.

The data of alpha and beta diversity concerning five subjects tested positive for different enteric pathogens (adenovirus, enterovirus, parechovirus, norovirus, *Campylobacter*, *Shigella*, and *G. duodenalis*) and four negative subjects were indicated in S3 (Table S3A, Figure S3B,D).

#### 3.3.4. Localities and Seasonality

The gut microbiota of patients sampled from semi-urban (Bonoua) and non-urban areas (Assouindé, Kimoukro, Yaou) showed no differences in alpha and beta diversity.

**Figure 3:**
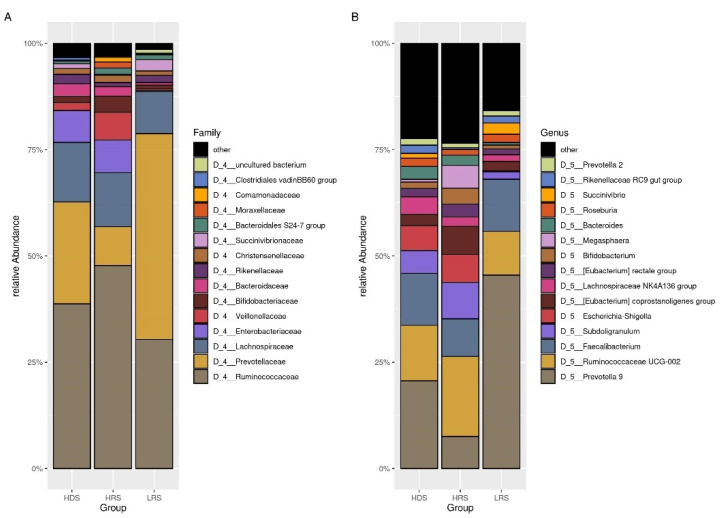
The plot of top 15 relative bacterial abundances. (**A**): the family level. (**B**): the genus level. The groups were based on the season of collection: the heavy dry season (HDS), heavy rainy season (HRS), and the low rainy season (LRS).

The relative abundances of the genus *Escherichia-Shigella* (0.00125 (0–0.00125)) in samples collected during the low rainy season was lower than in sample collected in the heavy dry season (0.0045 (0–0.59125)) and the heavy rainy season (0.003 (0–0.272)). The genus *Prevotella* 9 showed a significant lower abundance in the heavy rainy season (0.001375 (0–0.6885)) versus heavy dry season (0.05575 (0–0.7405)) and versus low rainy season (0.52625 (0.022–0.86675)), and in heavy dry season versus low rainy season. The genus *Megasphaera* showed a significant lower abundance in heavy dry season (0 (0–0.121)) versus the heavy rainy season (0 (0–0.6535)). The top 15 bacterial composition at the family and genus levels were shown in Figure 3.

### 3.4. Blastocystis, Commensal Entamoeba spp., and Intestinal Pathogens

Subjects negative for common enteric pathogens (xTAG Luminex panel), EV, PeV, AdV (real-time PCR), *Blastocystis*, and *Entamoeba* spp. (PCR) were assumed as the control group (Figure S3A,C). The other groups contained positive individuals who tested positive for one or more of these microorganisms. Only five individuals aged 5 to 10 years (4F/1M, 7.8%) were found to have multiple infections and exclusively reported frequent abdominal pains (Table S4A). The beta diversity analysis showed a significant lower abundance of the genus *Ruminococcaceae* UCG-002 in negative (0.0116 [0–0.082]) versus positive individuals (0.065 [0–0.8272]) (*p* < 0.01). The opposite trend for the genus *Faecalibacterium* does not reach significance.

**Figure 4:**
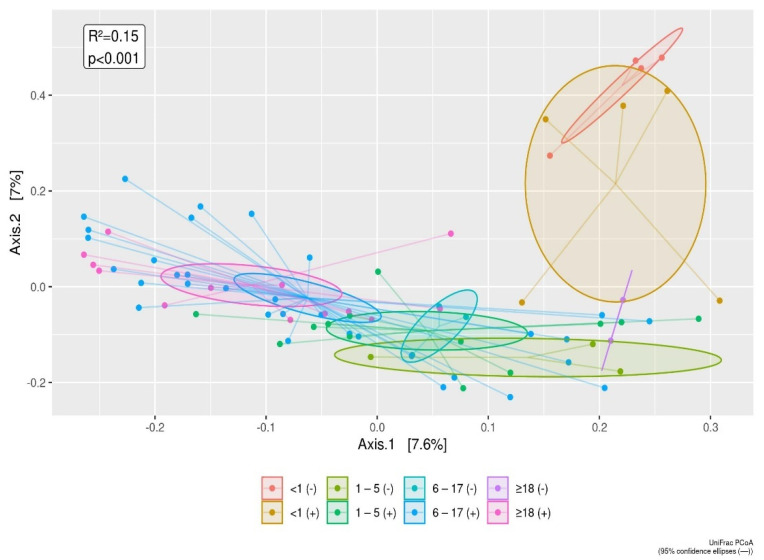
The UniFrac distances between enteric pathogen positive versus enteric pathogen negative subjects is displayed by age groups, using principal coordinate analysis (PCoA). The infant group tends to separate out according to Axis 1 (7.6%) and Axis 2 component (7%). The differences of the infant group from other age groups as shown in Figure 1, are also confirmed for comparisons of enteric pathogen negative versus enteric pathogen positive subjects.

No difference was found between positive versus negative subjects for the different age groups (Figure 4).

### 3.5. Blastocystis

To shed light on a possible implication of single or co-detection *Blastocystis* on gut microbiota, the control group (Group 1) was compared with 42 positive *Blastocystis* patients further divided as follows: 6 subjects positive for *Blastocystis* only (Group 2); 12 subjects positive for *Blastocystis* and *Entamoeba* spp. only (Group 3); 5 subjects positive for *Blastocystis*, viruses, and bacteria (Group 4a); 6 subjects positive for *Blastocystis* and *Entamoeba* spp., viruses and bacteria (Group 4b); 13 positive subjects for *Blastocystis* and *Entamoeba* spp. and *G. duodenalis* and other pathogens (Group 5b). Alpha diversity indices increased when *Entamoeba* spp. co-infections were present ( Table S4B).

**Figure 5:**
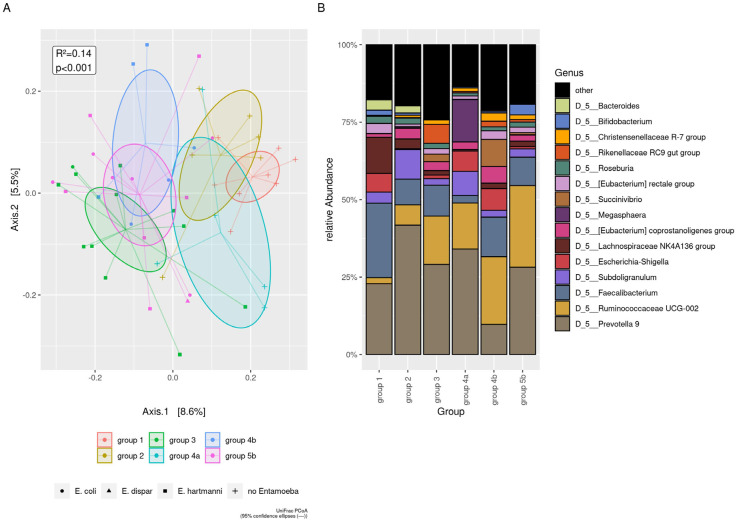
Beta diversity analysis in presence of *Blastocystis.* Group1: control group; Group 2: positive only for *Blastocystis*; Group 3: positive for *Blastocystis* and *Entamoeba* spp.; Group 4a: positive for *Blastocystis* and pathogenic virus and bacteria; Group 4b: positive for *Blastocystis* and *Entamoeba* spp. and pathogenic virus and bacteria; Group 5b: positive for *Blastocystis* and *Entamoeba* spp. and *G. duodenalis* and pathogenic virus, and bacteria. (**A**) The UniFrac distances among groups, using principal coordinate analysis (PCoA). UniFrac showed low divergences along Axis 1 (8,6%) between people carrying *Blastocystis* and *Entamoeba* spp. (Groups 3, 4b, 5b) compared to the negative subjects (Group 1) and subjects carrying *Blastocystis* but not *Entamoeba* spp. (Groups 2 and 4a). (**B**) The plot of top 15 relative bacterial abundances at the genus level.

As for bacterial composition, significant differences were observed between Group 1 versus Groups 3, 4a, 4b, and 5b but not versus subjects positive for *Blastocystis* only (Group 2). In detail, at the genus level, Group 1 showed: (i) a lower median of *Alloprevotella* (*p* < 0.01), *Rikenellaceae* RC9, *Succinivibrio* (*p* < 0.05), and *Ruminococcaceae* UCG-010 (*p* < 0.01) versus Group 3, (ii) a higher median of *Faecalibacterium* (*p* < 0.05) versus Group 4a; (iii) a lower median of *Ruminococcaceae* UCG-002 and *Ruminococcaceae* UCG-010 versus Group 4b; (iv) a higher median of *Bacteroides* (*p* < 0.05) and *Lachnoclostridium* (*p* < 0.05) and a lower median of *Ruminococcaceae* UCG-010 (*p* < 0.05) compared with Group 5b. Finally, Group 2 showed an increase of *Alloprevotella* and *Prevotella* 2 (*p* < 0.05) versus Group 3 (Figure 5). 

### 3.6. Commensal Entamoeba spp.

**Figure 6:**
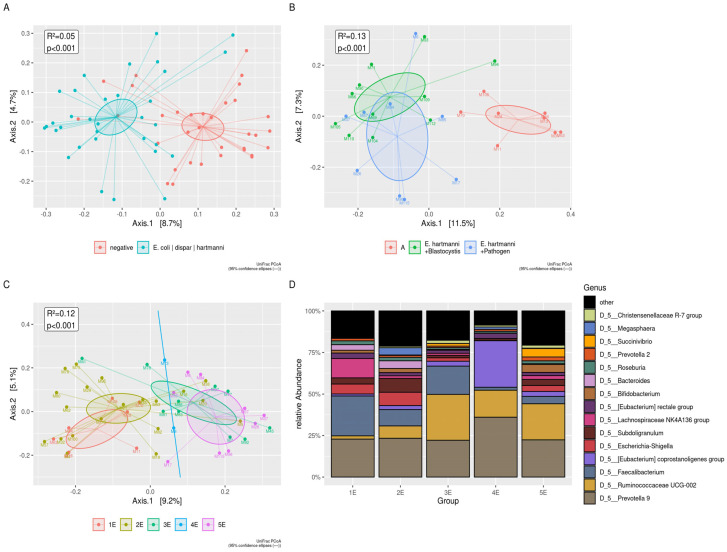
Beta diversity analysis in presence of *Entamoeba* spp. (**A**) The UniFrac distances between negative and positive subjects for *Entamoeba* spp., using principal coordinate analysis (PCoA) (**B**) The UniFrac distances among negative group, subjects harboring *E. hartmanni* and *Blastocystis*, and subjects harboring *E. hartmanni, Blastocystis*, and other pathogens, using principal coordinate analysis (PCoA). Group 1E: control group; Group: 2E: positive for pathogens not including *Entamoeba* spp.; Group 3E: subjects positive for *En. coli* and other pathogens; Group 4E: subjects positive for *E. dispar* and other pathogens; Group 5E: subjects positive for *E. hartmanni* and other pathogens. (**C**) The UniFrac distances among Group 1E, 2E, 3E, 4E, and 5E using principal coordinate analysis (PCoA). The groups carrying *En. coli, E. dispar,* and *E. hartmanni* (Groups 3E, 4E, and 5E)*,* clearly were separated from groups not harboring *Entamoeba* species (Groups 1E and 2E). (**D**) The plot of top 15 relative bacterial abundances at the genus level. All the significances relating to (**A**–**D**) are summarized in Table S5D.

*Entamoeba* spp. was isolated in 49% (33/67) subjects, mostly associated with *Blastocystis* (94%, 31/33). Firstly, we compared *Entamoeba* spp. negative versus *Entamoeba* spp. positive subjects (Figure 6A and Table S5A and Table S5B). To better elucidate the association between *Entamoeba* and *Blastocystis*, we then categorized *Entamoeba* spp. negative (*n* = 34) as follows: (i) a group carrying *Blastocystis* (*n* = 16), (ii) a group without *Blastocystis* (*n* = 18), and both groups were compared with patients harboring *Entamoeba* spp. with/without *Blastocystis* (*n* = 33), regardless of other infecting microorganisms (data not shown). To better clarify the frequently detected association between *E. hartmanni and Blastocystis* we distinguished the following groups: (i) the control group (*n* = 8), (ii) a group carrying *E. hartmanni* and *Blastocystis* (*n* = 11), (iii) a group carrying *E. hartmanni* and *Blastocystis* and pathogens (*n* = 9).

The presence of *E. hartmanni* produced a significant increase in alpha diversity indices (Table S4C). The UniFrac distances, using principal coordinate analysis (PCoA), showed the shifting of positive groups for *E. hartmanni* (Figure 6B). At the genus level of the *Christensenellaceae* R-7 group, *Ruminococcaceae* UCG-002 increased in two groups carrying *E. hartmanni*; *Blastocystis*, *Faecalibacterium*, and *Bifidobacterium* showed an opposite trend (Table S5C).

The next step was to discriminate any differential relationship between the control group (Group 1E) and the subjects infected by pathogens, not including *Entamoeba* spp. (Group 2E), or infected by different *Entamoeba* species (Group 3E = positive for *En. coli* and other pathogens; Group 4E = positive for *E. dispar* and other pathogens; Group 5E = positive for *E. hartmanni* and other pathogens). As for alpha diversity, a significant difference was found in Chao1 Index, Observed OTUs (*p* < 0.05) and Faith’s phylogenetic diversity (*p* < 0.01), comparing Group 1E versus Group 5E; observed OTUs and Faith’s phylogenetic diversity (*p* < 0.05) comparing Group 2E versus Group 5E (Table S4D).

Beta diversity analysis showed: (i) a lower abundance for *Bacteroides* (*p* < 0.05) and for *Lachnoclostridium* (*p* < 0.01) in Group 3E versus Groups 1E and 2E; (ii) a lower abundance for [*Ruminococcus*] torques group in Group 3E versus Group 2E; (iii) an increased abundance for *Ruminococcaceae* UCG-002 and *Ruminococcaceae* UCG-010 (*p* < 0.05) in Group 5E versus Group 2E, and for *Ruminococcaceae* UCG-010 (*p* < 0.01) in Group 5E versus Group 1E (Figure 6C,D).

### 3.7. G. duodenalis and Other Intestinal Pathogens

To investigate the influence of *G. duodenalis* and other enteropathogens, the 67 subjects were divided into seven groups: control group (Group A); 6 subjects positive for *Blastocystis* only (group C); 11 subjects positive for *Blastocystis* and *E. hartmanni* without enteric pathogens (group D); 6 subjects infected by *E. hartmanni*, *Blastocystis* and *G*. *duodenalis*, and enteric pathogenic bacteria and viruses (group E1); 6 subjects positive with *En. coli*, *Blastocystis* and *G. duodenalis*, and enteric pathogenic bacteria and viruses (group E2); 8 subjects positive with *Blastocystis*, *G. duodenalis,* and enteric pathogenic bacteria and viruses (group E3); 5 subjects positive for *Blastocystis* and enteric pathogenic bacteria and viruses (group M).

Observed OTUs, Faith’s PD, and Shannon index were significantly increased in group D compared to the control group and only for the Shannon index compared to group M (Table S4E).

**Figure 7:**
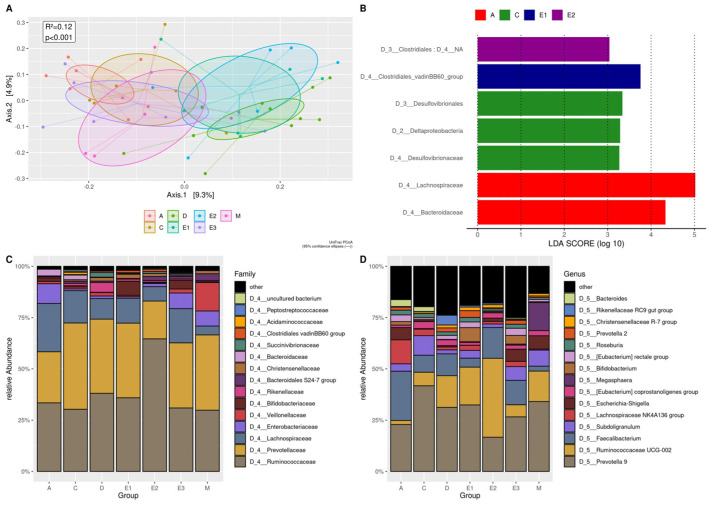
Beta diversity analysis for the subjects positive for *G. duodenalis* and other intestinal pathogens. Groups A: control group; Groups C: positive for *Blastocystis* only; Groups D: positive for *Blastocystis* and *E. hartmanni* without other pathogens; Groups E1: positive for *E. hartmanni, Blastocystis* and *G. duodenalis*, and pathogenic bacteria and viruses; Groups E2: positive for *En. coli, Blastocystis* and *G. duodenalis*, and pathogenic bacteria and viruses; Groups E3: positive for *Blastocystis, G. duodenalis*, and pathogenic bacteria and viruses; Groups M: positive for *Blastocystis* and mixed infection of pathogenic bacteria and viruses, without *G. duodenalis* and *Entamoeba* spp. (**A**) PCoA UniFrac showing low divergences along Axis 1 (9,3%) between positives with *Blastocystis* and *E. hartmanni* without pathogens (Group D) and Groups E1 and E2 including positives for *G. duodenalis* and other intestinal microorganisms. (**B**) Histogram of the linear discriminant analysis (LDA scores indicate the higher difference between clades of A, C, E1, and E2 groups. (**C**) The plot of top 15 relative bacterial abundances at the family level. (**D**) The plot of top 15 relative bacterial abundances at the genus level.

The UniFrac PCoA analysis along axis 1 showed some overlap in the composition of the groups and a non-significant separation between the three groups co-infected with *Entamoeba* species (D, E1, E2) and the other groups (A, E3, M) (Figure 7A); negative subjects (group A) were separated from subjects co-infected with *Entamoeba* spp. (D, E1, E2) as displayed in Figure 6A. Cladograms generated by LEfSe showed differences in taxa between A, C, E1, E2 groups with LDA score (*p* < 0.01) (Figure 7B). No significant differences between all groups were observed by the analysis of the relative abundance at the family and genus level (Figure 7C,D).

## 4. Discussion

Age, diet, mode of birth and lactation, environment, circadian rhythm, geographic location, and host genetics, in a complex way, contribute to the individual heterogeneity of the human gut microbiota [1]. The data currently available are mainly from populations living in so-called “Western” contexts such as Europe and North America where Bacteroides dominate the gut microbiota. Limited data are available from sub-Saharan rural or semi-urban areas where a predominance of Prevotella in bacteria composition is evidenced. However, the diffusing industrialization and westernization in these regions could influence these patterns [8,34,35].

The present study describes the composition of the intestinal microbiota in infants, children, and adults living in the Côte d’Ivoire without neglecting the most common endemic, pathogenic, and commensal microorganisms. This investigation aimed to delineate a potential link between *Blastocystis*, commensal *Entamoeba* spp., common enteric pathogens, and the gut microbiota [36,37]. Among adults, two clusters, one dominated by Ruminococcaceae and the other one by Prevotellaceae, could be identified. These species could be either termed “biomarkers” as proposed by Gorvitovskaia et al. [38], or “biomes types” as indicated by Huse et al. [39]. These species represent consortia of bacteria mostly influenced by genetic and immune host factors, and, above all, dietary and cooking practices [40]. The reduced abundance of Bacteroidaceae, in both clusters, was in line with previous data from similar eco-geographical areas, from agricultural societies, and countries with a low level of industrialization [10,41]. In our sampling area, weaning occurs earlier than in Western societies and the diet mostly includes local and seasonal vegetables (e.g., okra, cassava and cassava leaf, tubers, and plantain) prevailing on the intake of animal-based product (e.g., eggs, poultry, fish). Among the fats, peanuts, and palm oil dominate. Seasonal fruit consumption is not a widespread habit, due to economic reasons and its rapid deterioration and traditional sugary juices (pineapple, passion fruit, hibiscus, ginger) are preferred, therefore reducing the intake of vitamins, mineral salts and healthy fiber. Overall, the present investigation confirmed the different composition of gut microbiota between infants, children, and the high individual variation among adults [4,42]. Enterobacteriales (Proteobacteria phylum), Bifidobacteriales (Actinobacteria phylum), and Lactobacillales (Firmicutes phylum) showed higher relative abundance in infants, while Firmicutes and Bacteroidetes were the dominant phyla in older individuals, followed by Proteobacteria [43,44]. In all age groups, *Faecalibacterium*, *Lachnospiraceae*, *Eubacterium rectale*, *Roseburia*, members of Clostridium clusters XIVa and IV, have been detected. These taxa produce crucial metabolites that promote health and maintain gut microbiome colonization resistance [45]. The lack of complete uniformity with previous studies could be attributable to regional and geographical differences [10,46,47,48].

Regarding sex-related microbiota differences, females showed a higher phylogenetic diversity than males. It is possible to hypothesize that puberty in young women could have an impact on alpha diversity compared to males. Additionally, ethnic and social habits related to daily activities could contribute to an exposition of men and women to different environmental factors [49]. A sex-related divergence of *Prevotella* 9, *Ruminococcaceae* UCG-002, and *Megasphaera* was noted in different age groups. In the present cohort, *Megasphaera* was almost undetectable in both sexes, except in women >18 years of age, which is different from the study by Takagi et al. [50], who reported high levels in Japanese adult males. The meaning of these differences will require further detailed investigations.

A specific aim of the study was to provide new data on the microbiota composition in subjects harboring *Blastocystis*, *Entamoeba* spp., and common enteropathogens. The high detection of protozoa viruses and bacteria confirmed data reported by previous studies conducted in the same area [26,51] and other countries in sub-Saharan Africa [9,19,52,53,54]. No patients were tested positive for *E. histolytica*, *C. hominis*, and *C. parvum*. Unfortunately, the use of the xTAG GPP molecular test did not allow the isolation of *Cryptosporidium meleagridis* whose prevalence is relatively high in the same area [55].

The high number of asymptomatic individuals suggests an adaptation to constant and cumulative exposure to enteropathogens due to the common risk factors associated with low-income countries [22,23,56,57,58].

In children and adults carrying protozoa, bacteria, or viruses, greater diversity and richness in the gut microbiota than in negative subjects was evident, in line with other works [19,20]. Furthermore, the critical taxa involved in the intestinal homeostasis such as *Faecalibacterium*, *Bifidobacterium*, *Lachnospiraceae*, *Ruminococcaceae* UCG-002, (*Eubacterium*) *coprostanoligenes*, (*Eubacterium*) *rectale*, and *Roseburia* group showed variations in richness in the same comparison.

The variable abundance of bacterial genera such as *Faecalibacterium*, *Bifidobacterium*, *Roseburia* group allows for the production of key metabolites as short-chain fatty acids (SCFA), e.g., butyrate and succinate, lactate, or acetate to preserve homeostasis [1,4,59]. This scenario could reflect a bidirectional ability of adaptation between the host’s immune response and bacterial composition. *Faecalibacterium prausnitzii* and *E. rectale* are responsible for a major fraction of butyrate production and consequently play a crucial role in shaping the local and peripheral immune system. The emerging evidence of diet-induced changes in SCFA-producing bacteria, as reported by Morrison et al. [60], could suggest a possible contribution of Ivorian eating habits in the modulation of adaptive immunity.

*Blastocystis* was highly prevalent in our cohort and a non-significant variation of alpha diversity between *Blastocystis* positive-only group and the control group is evidence of the modest effect that *Blastocystis* carriage has [61,62]. The lower abundance of Bacteroides in subjects infected with *Blastocystis* seems to confirm the hypothesis of a minor predisposition to harbor this parasite in “Bacteroides-driven enterotype” subjects, as described for Danish and Spanish individuals by O’Brien Andersen et al. [63], and Mexican subjects by Nieves-Ramirez [64]. In all groups carrying *Blastocystis*, bacterial genera associated with a healthy status, such as *E. rectale* and *coprostanoligenes* groups, *Roseburia* and *Succinivibrio* showed a constant presence, although with a reduced relative abundance versus control group [46,65,66,67,68]. These specific OTUs could explain a beneficial influence on normobiosis [61] also in conjunction with a traditional African diet. Considering the genetic diversity within *Blastocystis*, with at least 17 different subtypes, the possible implications in the pathophysiology of human infections related to the different subtypes should be investigated to better understand the role of this parasite on the human gut microbiota [63,69].

Concerning commensal *Entamoeba* spp., an increase in alpha diversity in subjects co-harboring *Entamoeba* spp. and *Blastocystis*, especially in case of *E. hartmanni* co-occurrence, was evidenced. Clear separation in Unifrac distance was found and the increase of *Succinivibrio*, *Christensenellaceae* R-7 group, *Ruminococcaeae* UCG-002, *Rikenellaceae* RC9 intestinal group seem associable with *Entamoeba* perturbations. Bacterial niches, responsible for beneficial or detrimental associations in subjects positive for *Blastocystis* alone or co-harbored with commensal *Entamoeba* spp., should be further investigated [70,71]. The increase in *Succinivibrio* in people detected positive for *E. hartmanni*, *Blastocystis*, and pathogens seems to confirm the data reported from Cameroon by Morton et al. (19) and in Malawian children by Ordiz et al. [72]. These data suggest that recurrent endemic intestinal infections in rural countries [73], together with eating habits [10] could favor, in some groups of African natives compared to African Americans, the conditions for a stable presence of *Succinivibrio* in the gut microbiota. The low number of subjects harboring *G. duodenalis* and pathogenic viruses and bacteria precluded the statistical significance in any comparisons. However, an interesting aspect seems to emerge: some protective taxa (e.g., *E. rectale* group, *Roseburia*, *Christensenellaceae*) showed an expansion despite the reduction of *Faecalibacterium*, as previously reported [46,74,75,76,77,78]. The relative abundances of *Bifidobacterium* and *Faecalibacterium* were oppositely related. Subjects containing multiple enteric pathogens, without *G. duodenalis* and *Entamoeba* spp., showed a decrease in so-called protective taxa (*Bifidobacterium* and *Faecalibacterium*) while the prevalence of *Megasphaera* increased. This result should be better investigated concerning the potential involvement of *Megasphaera* in the adaptability of the human gut ecosystem [72,79,80].

## 5. Conclusions

In conclusion, the co-occurrence of *Blastocystis* and commensal *Entamoeba* spp., despite the presence of other enteric pathogens including *G. duodenalis*, seems to preserve a high diversity, favor different bacterial consortia, and does not compromise the intrinsic ability of intestinal microbiota to restore and/or maintain homeostasis [74,81,82,83,84,85]. However, the beneficial or potential regulatory effect of *Blastocystis* is reduced in subjects positive for bacterial and viral pathogens without *G. duodenalis* and commensal *Entamoeba* spp., probably reducing the entity of symptoms.

Finally, the complex scenario observed in this cohort from Côte d’Ivoire helps to provide an integrated view of human gut microbiota composition. Simultaneous testing of common intestinal pathogens supplies new data from Africa, still underrepresented in the global picture of the gut microbiota, and helps to better understand the balances of intestinal homeostasis.

Future investigations are needed to verify if the influence of rural diet on gut microbiota can play a role in seasonal predisposition to endemic enteritis. It will be very important to extend the understanding of eukaryotic microorganisms’ interactions in the gut microbiota in health status and intestinal infections.

## Figures and Tables

**Table 1:** Demographic characteristics of enrolled individuals.

	Age Groups	<1 year		>1–5 years		6–17 years		>18 years		Total	
Localities		*n* F/M	Median min–max	*n* F/M	Median min–max	*n* F/M	Median min–max	*n* F/M	Median min–max	*n* F/M	Median min–max
Bonoua		8 2/6	0.1 0.02–0.5	10 6/4	4.5 2–5	20 10/10	11.5 8–17	15 7/8	30 18–59	53 25/28	11 0.02–59
Assouindé		1 1/0	0.75	-	-	3 2/1	11 11–15	-	-	4 3/1	11 0.75–15
Kimokro		-	-	1 0/1	2.5	5 2/3	8 6–10	-	-	6 2/4	7.5 2.5–10
Yaou		-	-	5 3/2	5 4–5	8 3/5	6.5 6–13	-	-	13 6/7	6 4–13
Total		9 3/6	0.1 0.1–0.7	16 9/7	4.5 1.8–5	36 17/19	10 6–17	15 7/8	0.1 0.1–05	76 36/40	9 0.02–59

**Table 2:** Summary of detected bacterial and viral pathogens.

Viruses and Bacteria	Positive Samples *n* (F/M)	Mean Age (min–max)	Detected Pathogens (*n*)
Adenovirus (AdV)	17 (9/8)	13.5 (0.25–59)	EV (2); NoV (2); ETEC (3); *Campylobacter* (1)
Parechovirus (PeV)	4 (1/3)	5.4 (0.1–14)	EV (2); NoV (1); *Campylobacter* (1)
Enterovirus (EV)	14 (5/9)	6.5 (0.1–14)	PeV (2); AdV (3); NoV(3); *Campylobacter* (2); ETEC (4)
Norovirus G1/G2 (NoV)	8 (3/5)	12.7 (0.1–39)	EV (3); AdV (2); PeV (1); RoV (1)
Rotavirus A (RoV)	1 (1/0)	39	RoV (1)
*Campylobacter*	5 (3/2)	6.3 (9.75–9)	*E.coli* O157 (1); ETEC (1); STEC (1); *Shigella* (1); EV (2); PeV (1); AdV (1)
*E.coli* O157	4 (2/2)	11 (17–8)	ETEC (3); STEC (1); *Campylobacter* (1); *Shigella* (1); EV (2); PeV (1); AdV (1)
ETEC	10 (6/4)	7 (1.8–17)	*E.coli* O157 (3); STEC (2); *Campylobacter* (3); *Shigella* (2); EV (4); AdV (3)
*Salmonella*	1 (0/1)	43	-
STEC	2 (1/1)	9 (9)	*E.coli* O157 (1); ETEC (2); *Campylobacter* (1)
*Shigella*	4 (3/1)	11.7 (0.75–24)	*E.coli* O157 (1); ETEC (2); *Campylobacter* (1); PeV (1)

ETEC: enterotoxigenic *E. coli*, STEC: Shiga-like toxin producing *E. coli*.

**Table 3:** Protozoa distribution based on age and localities.

	*E. hartmanni*		*En. coli*		*E. dispar*		*Blastocystis*		*G. duodenalis*	
Localities	Sex	Age	Sex	Age	Sex	Age	Sex	Age	Sex	Age
	*n* (F/M)	Mean (min–max)	*n* (F/M)	Mean (min–max)	*n* (F/M)	Mean (min–max)	*n* (F/M)	Mean (min–max)	*n* (F/M)	Mean (min–max)
Bonoua	16 (6/10)	21 (9–50)	2 (2F)	3 (2–4)	1 (1F)	5	28 (17/11)	22 (9–59)	8 * (6/2)	17 (2–50)
Assouindé	1 (1F)	11	-	-	-	-	3 (2/1)	12 (11–15)	3 (2/1)	12 (11–15)
Kimoukro	1 (1M)	6	3 (1/2)	7 (2–10)	-	-	4 (2/2)	8 (6–10)	7 (2–10)	8 (4/4)
Yaou	2 (2F)	5 (4–6)	6 (4/2)	6 (5–7)	1 (1M)	5	12 (6/6)	6 (4–13)	8 (4/4)	5 (4–7)
Total	20 (9/11)	18 (4–50)	11 (7/4)	12 (2–59)	2 (1/1)	5	47 (27/20)	14 (4–59)	24 (14/10)	11 (2–50)

* One-month child was detected positive for *G. duodenalis.*

**Table 4:** Alpha diversity indices in the age groups.

	Age Groups (*n*)	<1 year (9) Median (min–max)	1–5 years (16) Median (min–max)	6–17 years (36) Median (min–max)	>18 years (15) Median (min–max)
Alpha Diversity					
Shannon index		1.703 (0.6059–3.019)	3.411 (2.2730–4.131) ^c^	3.703 (2.1940–4.962) ^a^	3.631 (1.5140–4.589) ^c^
Observed OTUs		39.00 (19.00–86.00)	151.00 (79.00–258.00) ^b^	189.00 (94.00–459.00) ^a^	177.00 (82.00–305.00) ^a^
Faith’s phylogenetic diversity		4.601 (0.9572–12.04)	12.850 (7.2710–20.82) ^c^	16.560 (7.3410–34.15) ^a^	17.540 (8.4570–25.67) ^a^
Chao1 index		39.50 (19.00–95.07)	189.50 (98.60–313.30) ^b^	228.20 (105.50–610.00) ^a^	231.00 (100.40–406.90) ^b^

^a^*p* < 0.001, ^b^ *p* < 0.01, ^c^ *p* < 0.05 versus <1 year.

## Data Availability

The dataset associated to this paper can be accessed on https://www.ncbi.nlm.nih.gov/sra/PRJNA741457 (accessed on 15 August 2021).

## Supplementary Materials

The following are available online at https://www.mdpi.com/article/10.3390/microorganisms9081763/s1, **Figure S1A.** The plot of top 15 bacterial relative abundances at the Family level in each age group; **Table S1A.** The top 15 bacterial relative abundances; **Figure S2**. Beta diversity of Cluster 1 versus Cluster 2 (Denovo clustering); **Table S3A**. Alpha diversity indices of positive and negative subjects, grouped in <1 year and >1 year; **Figure S3**. Positive versus negative subjects comparisons: Beta diversity analysis; **Table S4A**. All microorganisms detected in the 5 symptomatic subjects; **Table S4B.** Comparison of *Blastocistys* groups: Alpha diversity index; **Table S4C.** Comparison of *E. hartmanni* groups: Alpha diversity index; **Table S4D.** Comparison of *Entamoeba* species groups: Alpha diversity index; **Table S4E**. Comparison of *G. duodenalis* and other intestinal pathogens positives: Alpha diversity index; **Table S5A.** Beta diversity analysis for *Entamoeba* spp. presence; **Table S5B.** Beta diversity analysis for *Entamoeba* spp. presence; **Table S5C.** Comparison of *E. hartmanni* groups: Beta diversity at genus level; **Table S5D.** Beta diversity analysis for *Entamoeba* spp. detection; **Table S5E.** Beta diversity analysis for *Entamoeba* spp. presence.
