# Supplementary material for: Analysis of Human Gut Microbiota Composition Associated to the Presence of Commensal and Pathogen Microorganisms in Côte d’Ivoire
Source: Microorganisms. 2021 Aug 18;9(8):1763. doi: 10.3390/microorganisms9081763 (PMC8400437; doi:10.3390/microorganisms9081763)
Supplement: Supplementary file 1 [file microorganisms-09-01763-s001.zip › microorganisms-1330453-supplementary/Figure S1A and Table S1A.pdf]

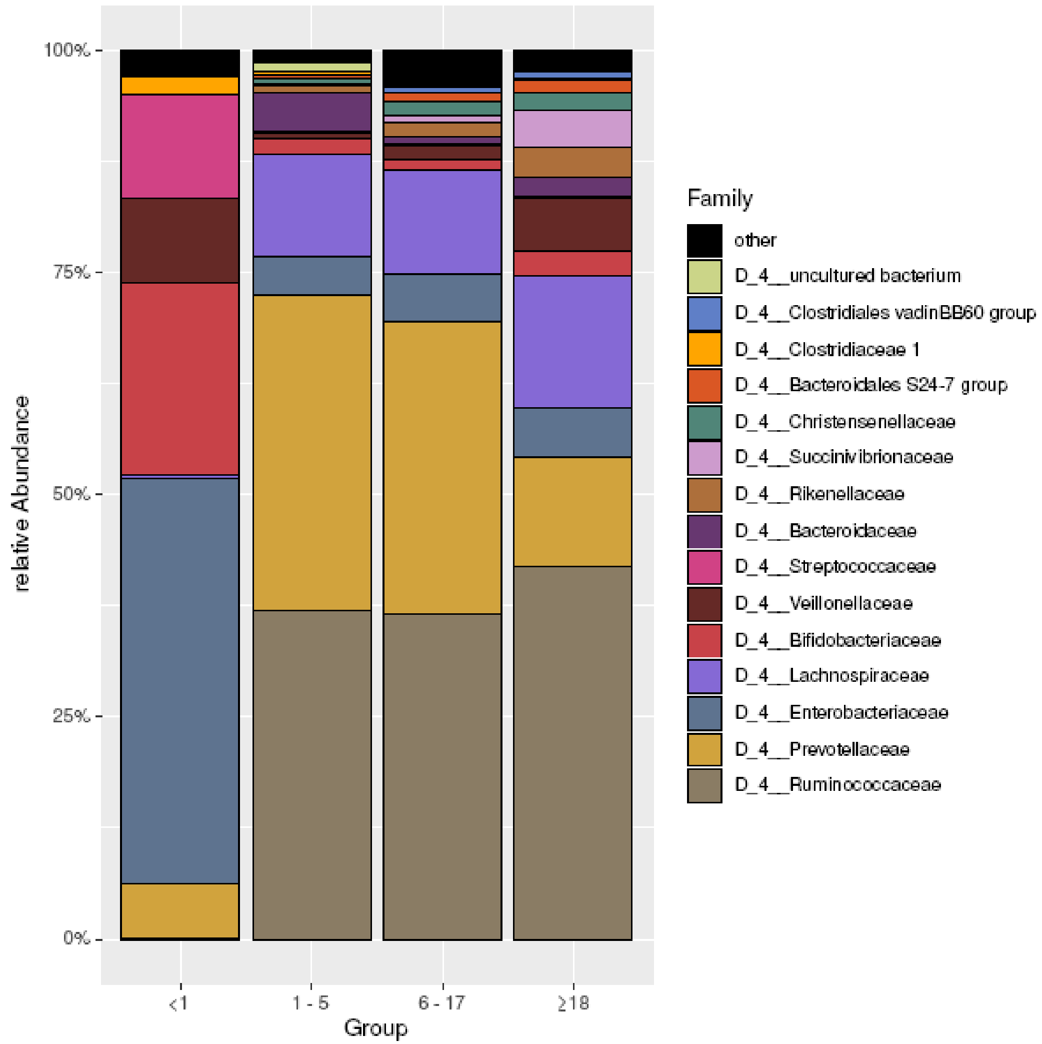

Figure S1A. The plot of top 15 bacterial relative abundances at the family level in each age group.

Table S1A. The top 15 bacterial relative abundances

Age group (n) / Top15 Genus abundance	**<1y (9) Median [min-max]**	**<1y versus >1 - 5y (16) p value**	**<1y versus >6 - 17y (36) p value**	**<1y versus >18y (15) p value**
[Eubacterium] coprostanoligenes group	0 [0-0]	0.0138	0.0007	0.0006
[Eubacterium] rectale group	0 [0-0]	0.0183	0.0091	0.0225
Bacteroides	0 [0-0.0048]	0.0171	0.0625	0.0362
Bifidobacterium	0.0222 [0-0.8670]	0.8661	1.0000	1.0000
Escherichia-Shigella	0.00125 [0-0.720]	0.3045	0.0512	0.0872
Faecalibacterium	0 [0-0.0013]	0.0005	0.0005	0.0362
Klebsiella	0 [0-0.0160]	0.0138	0.0030	0.0321
Lachnospiraceae NK4A136 group	0 [0-0]	0.0016	0.0007	0.0036
Megasphaera	0 [0-0,6535]	1.0000	1.0000	1.0000
Prevotella 9	0 [0-0.5423]	0.0165	0.0026	0.2495
Roseburia	0.0135 [0-0.0295]	0.0029	0.0011	0.0170
Ruminococcaceae UCG-002	0 [0-0.0003]	0.0165	0.0007	0.0006
Streptococcus	0 [0-0.0073]	0.5373	0.2866	0.3871
Subdoligranulum	0 [0-0.008]	0.0043	0.0007	0.0004
Veillonella	0 [0-0.0158]	0.4681	0.0606	0.1607

Comparison between the group <1 year and the other age groups, at the genus level.
p value by Kruskal Wallis test.
y means year
