# Supplementary material for: Analysis of Human Gut Microbiota Composition Associated to the Presence of Commensal and Pathogen Microorganisms in Côte d’Ivoire
Source: Microorganisms. 2021 Aug 18;9(8):1763. doi: 10.3390/microorganisms9081763 (PMC8400437; doi:10.3390/microorganisms9081763)
Supplement: Supplementary file 1 [file microorganisms-09-01763-s001.zip › microorganisms-1330453-supplementary/Figure S2.pdf]

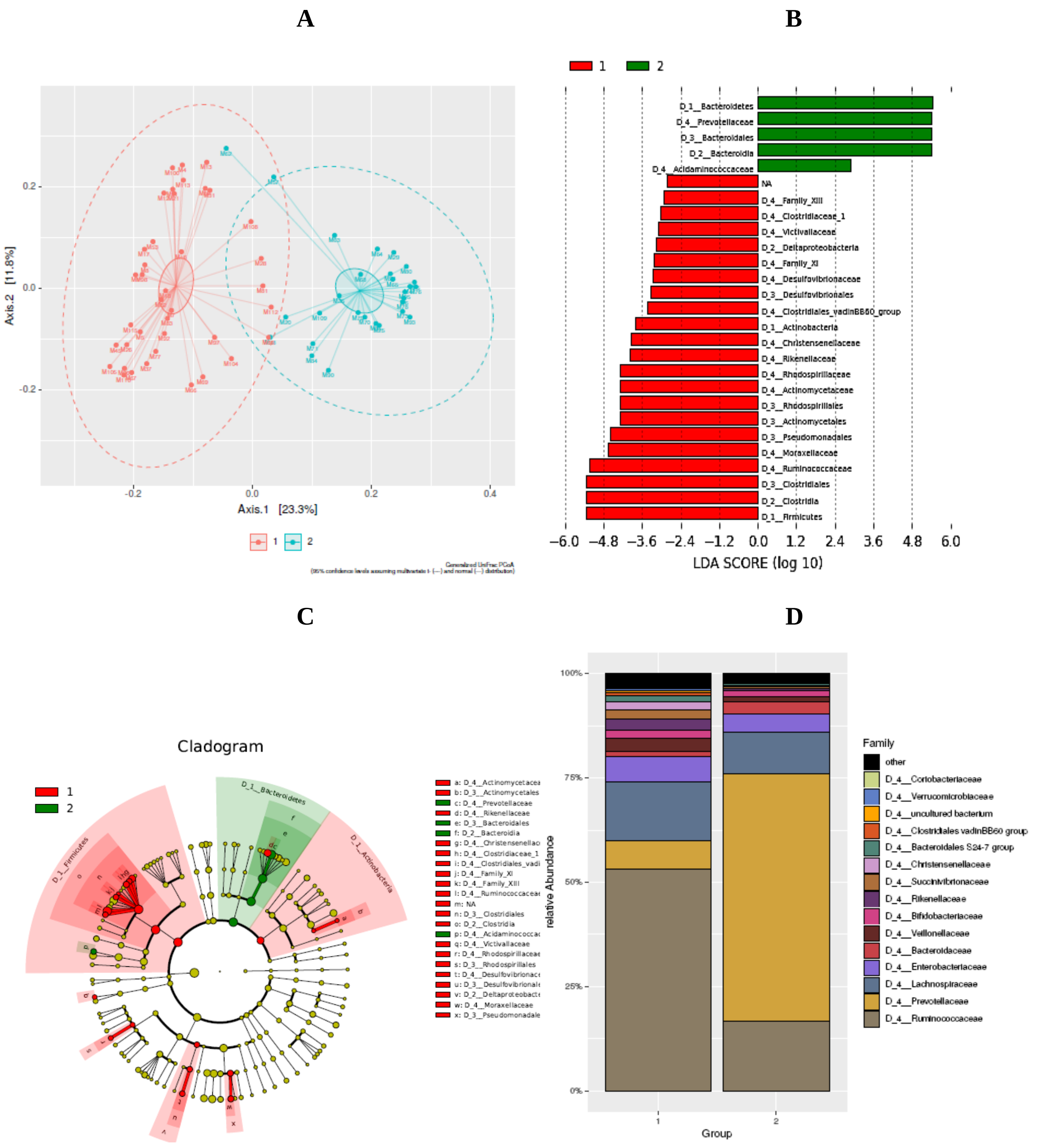

Figure S2. **Beta diversity of Cluster 1 versus Cluster 2 (Denovo clustering)**: (S2 FigA) The Unifrac distances between Cluster 1 (39 subjects) and Cluster 2 (28 subjects) using principal coordinate analysis (PCoA). (S2 FigB) The linear discriminant analysis (LDA). (S2 FigC) The effect size (LEfSe) analysis. S2 FigB and S2 FigC indicate the higher difference between clades of Cluster 1 and Cluster 2. (S2 FigD) The plot of top 15 relative bacterial abundances at the family level.
