# Supplementary material for: Analysis of Human Gut Microbiota Composition Associated to the Presence of Commensal and Pathogen Microorganisms in Côte d’Ivoire
Source: Microorganisms. 2021 Aug 18;9(8):1763. doi: 10.3390/microorganisms9081763 (PMC8400437; doi:10.3390/microorganisms9081763)
Supplement: Supplementary file 1 [file microorganisms-09-01763-s001.zip › microorganisms-1330453-supplementary/Table S3A and Figure S3.pdf]

Table S3A Alpha diversity indices of positive and negative subjects, grouped in <1year and >1year.

Variables	<1year	Median [min−max]	p value	>1year	Median [min−max]	p value
**Patients (number)**	Negative (4)	0.10 [0.02−0.17]	0.1271	Negative (8)	9.00 [3.00−30.00]	0.6389
	Positive (5)	0.25 [0.10−0.75]		Positive (59)	10.00 [1.80−59.00]	
**Chao1 index**	Negative (4)	30.25 [19.00−49.27]	0.1462	Negative (8)	150.70 [100.40−278.80]	0.0087
	Positive (5)	67.00 [20.00−95.07]	0.1462	Positive (59)	221.50 [98.60−610.00]	
**Observed OTUs**	Negative (4)	30.00 [19.00−46.00]	0.2359	Negative (8)	125.00 [82.00−201.00]	0.0037
	Positive (5)	46.00 [20.00−86.00]	0.2359	Positive (59)	179.00 [79.00−459.00]	
**Faith's phylogenetic diversity**	Negative (4)	2.259 [0.9572−12.040]	0.8066	Negative (8)	11.42 [8.457−15.23]	8.005E-05
	Positive (5)	4.654 [2.7080−8.697]	0.8066	Positive (59)	16.17 [7.271−34.15]	
**Pielou's evenness**	Negative (4)	0.5158 [0.3748−0.6605]	0.7370	Negative (8)	0.6546 [0.3435−0.7211]	0.1752
	Positive (5)	0.5549 [0.1764−0.6777]	0.7370	Positive (59)	0.7239 [0.4092−0.8355]	
**Shannon index**	Negative (4)	1.569 [1.3800−2.420]	0.8576	Negative (8)	3.138 [1.514−3.817]	0.0760
	Positive (5)	1.887 [0.6059−3.019]	0.8576	Positive (59)	3.636 [2.194−4.962]	

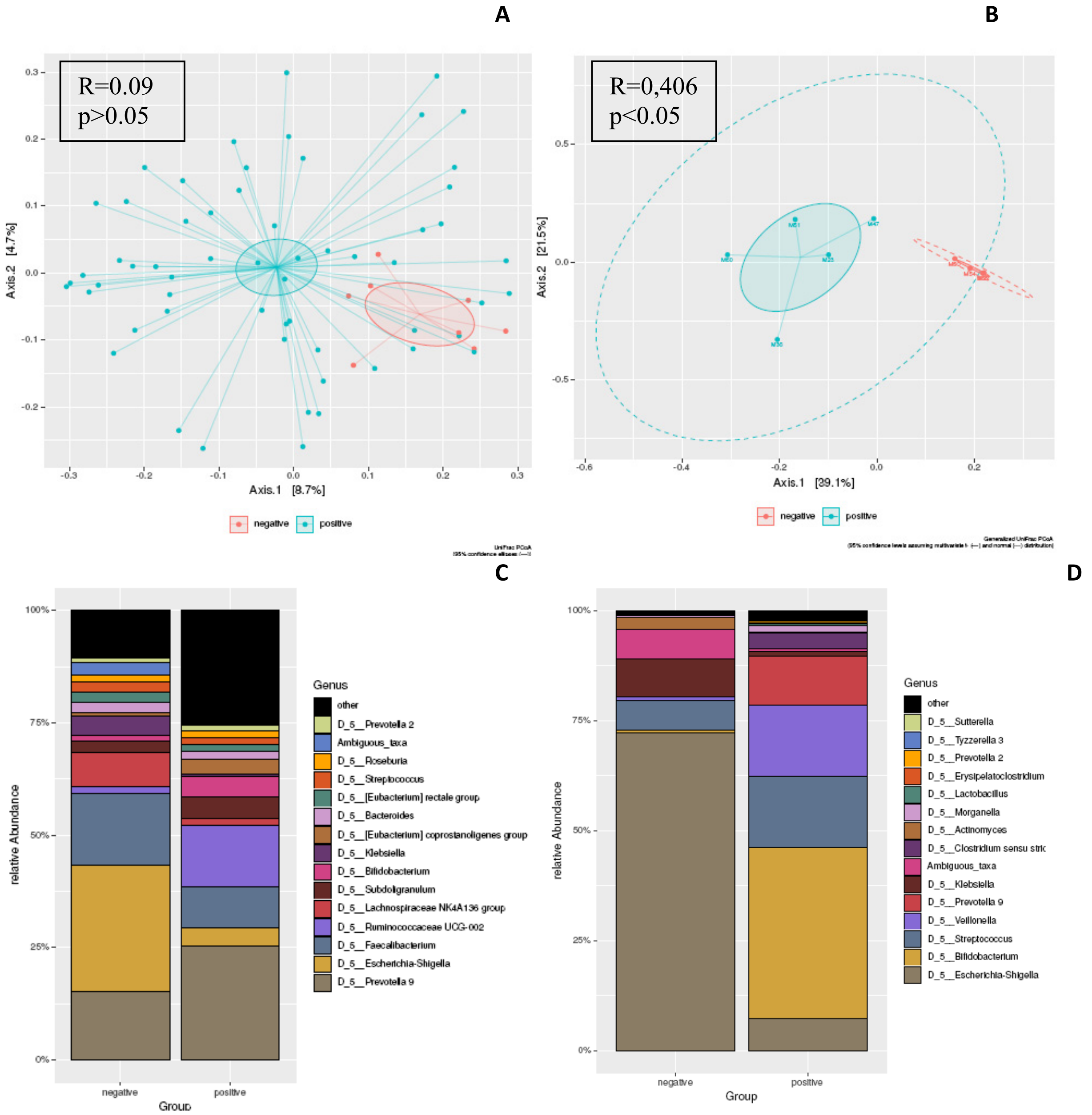

Figure S3. Positive versus negative subjects comparisons: Beta diversity analysis. (S3 FigA): The UniFrac distances between positive (n=59) and negative (n=8) subjects (>1 year) using principal coordinate analysis (PCoA). (S3 FigB) The UniFrac distances between positive(n=5) and negative (n=4) subjects (< 1 year). (S3 FigC) The plot of top 15 relative bacterial abundances at the Genus level, showing a diversity of positive versus negative in > 1 year subjects (S3 FigD) The plot of top 15 relative bacterial abundances at the genus level, showing a diversity of positive versus negative in <1 year subjects.
