# Supplementary material for: Analysis of Human Gut Microbiota Composition Associated to the Presence of Commensal and Pathogen Microorganisms in Côte d’Ivoire
Source: Microorganisms. 2021 Aug 18;9(8):1763. doi: 10.3390/microorganisms9081763 (PMC8400437; doi:10.3390/microorganisms9081763)
Supplement: Supplementary file 1 [file microorganisms-09-01763-s001.zip › microorganisms-1330453-supplementary/Table S4A-E.pdf]

Table S4A. All microorganisms detected in the 5 symptomatic subjects

Age (years)	Sex	Luminex panel pathogens *Blastocystis and Entamoeba* spp.	Symptoms
5	F	ETEC, *Shigella, G. duodenalis*	Frequent abdominal pains
8	F	*E.coli* O157, *G. duodenalis*, *Blastocystis*	Frequent abdominal pains
6	M	*Campylobacter*, ETEC, adenovirus, enterovirus, *G.duodenalis, Blastocystis, En. hartmanni*	Frequent abdominal pains
10	F	*E.coli* O157, ETEC, *G.duodenalis, Blastocystis, En. coli*	Frequent abdominal pains
5	F	*Campylobacter*, ETEC, *G.duodenalis, En. dispar*	Frequent abdominal pains

Abbreviations: ETEC means enterotoxigenic *E. coli*

Table S4B. Comparison of *Blastocistys* groups: Alpha diversity index

Groups (n) Median [min-max] / Alpha Diversity index	Group 1 (8) Control group	Group 2 (6) *Blastocystis* positive only	Group 3 (12) *Blastocystis*+ *Entamoeba* spp.	Group 4a (5) *Blastocystis*+ virus +bacteria	Group 4b (6) *Blastocystis*+ *Entamoeba* spp. +virus+bacteria	Group 5b (13) *Blastocystis*+ *Entamoeba* spp. +virus+bacteria +*G. duodenalis*
Chao1	150.70 [100.40-278.80]	217.60 [140.20—231.00]	301.10 [126.60—454.70]*	154.20 [105.50—209.90]§	300.30 [156.00—383.20]	238.10 [172.00—610.00]
Observed OTUs	125.00 [82.00—201.00]	173.50 [117.00—189.00]	248.00 [122.00—348.00]**	130.00 [94.00—170.00]§	238.00 [141.00—290.00]	189.00 [160.00–459.00]
Faith's Phylogenetic Diversity	11.42 [8.457—15.23]	14.37 [11.460—25.67]	21.80 [8.925—29.68]**	13.63 [7.372—15.67]	19.71 [15.940—23.98]*	17.82 [11.360—34.15]*
Pielou's evenness	0.6546 [0.3435—0.7211]	0.6951 [0.5291—0.7336]	0.7310 [0.4513—0.8301]*	0.5929 [0.4513–0.7169]§	0.7243 [0.5304—0.7552]	0.7282 [0.4092—0.8221]
Shannon	3.138 [1.514—3.817]	3.586 [2.520—3.845]	4.223 [3.699—4.589]**	3.045 [2.315-3.308]§§	3.707 [2.939—4.282]	3.770 [2.194—4.962]

Group1: negative subjects; Group 2: positive only for *Blastocystis*; Group 3: positive for *Blastocystis*and *Entamoeba* spp.; Group 4a: positive for *Blastocystis* and pathogenic virus and bacteria; Group 4b: positive for *Blastocystis* and *Entamoeba* spp. and pathogenic virus and bacteria; Group 5b: positive for *Blastocystis* and *Entamoeba* spp. and *G, duodenalis* and pathogenic virus and bacteria.

*means p < 0.05, **means p < 0.01, in comparison of group 1versus other groups.§means p < 0.05, §§means p < 0.01, in comparison of Group 4a versus Group 3 (Kruskal-Wallis test).

Table S4C. Comparison of *En. hartmanni* groups: Alpha diversity index

Groups (n) Median [min-max] / Alpha Diversity index	Control group (8)	*En. hartmanni* +*Blastocystis* group (11)	*En. hartmanni*+*Blastocystis* +Pathogen group (9)
Chao1	150.70 [100.40 − 278.80]	271.90 [126.60 − 454.70]	324.40 [172.00 − 547.60]
Observed OTUs	125.00 [82.00 − 201.00]	243.00 [122.00 − 336.00]	258.00 [160.00 − 437.00]
Faith's Phylogenetic Diversity	11.42 [8.457 − 15.23]	21.39 [8.925 − 27.77]	21.62 [14.080 − 28.96]
Pielou's evenness	0.65 [0.3435 − 0.7211]	0.78 [0.6648 − 0.8355]	0.73 [0.5304 − 0.8221]
Shannon	3.14 [1.514 − 3.817]	4.25 [3.699 − 4.589]	4.10 [2.939 − 4.833]

Control group versus Group of subjects positive for *En. hartmanni and Blastocystis*, and versus Group of subjects positive for *En. hartmanni and Blastocystis* andother pathogens.

* means $p < 0.05$ and ** means $p < 0.01$ Control group versus *En. hartmanni and Blastocystis*; ° means $p < 0.05$ and °° means $p < 0.01$ Control group versus *En. hartmanni and Blastocystis and* pathogens; (Kruskal-Wallis test).

Table S4D. Comparison of *Entamoeba* species groups: Alpha diversity index

Groups (n) Median [min-max] / Alpha Diversity index	Group 1E (8) Control group	Group 2E (20) *Entamoeba* spp. Negative +Pathogens	Group 3E (10) *En. coli* +Pathogens	Group 4E (2) *En. dispar* +Pathogens	Group 5E (9) *En. hartmanni* +Pathogens
Chao1	150.70 [100.40−278.80]	193.60 [105.50−356.70]	228.80 [98.60−610.00]	151.10 [118.10−184.10]	324.40 [172.00−547.60]*
Observed OTUs	125.00 [82.00−201.00]	148.50 [79.00−324.00]§	188.00 [95.00−459.00]	136.50 [111.00−162.00]	258.00 [160.00−437.00]*§
Faith's Phylogenetic Diversity	11.42 [8.457−15.23]	13.23 [7.341−26.59] §	17.79 [7.271−34.15]	12.66 [11.360−13.95]	21.62 [14.080−28.96]**§
Pielou's evenness	0.6546 [0.3435−0.7211]	0.7016 [0.4513−0.8301]	0.7243 [0.4092−0.8095]	0.5906 [0.4952−0.6860]	0.7299 [0.5304−0.8221]
Shannon	3.138 [1.514−3.817]	3.314 [2.234−4.562]	3.604 [2.194−4.962]	2.911 [2.332−3.490]	4.105 [2.939−4.833]

Groups 1E (negatives) versus other groups. Group 1E: negative subjects; Group: 2E: positive for pathogens not including *Entamoeba* spp.; Group 3E: subjects positive for *En. coli and*other pathogens; Group 4E: subjects positive *En. dispar* andother pathogens; Group 5E: subjects positive for *En. hartmanni* andother pathogens.

* means $p < 0.05$, ** means $p < 0.01$ in comparison the groups 1E versus 5E; §means $p < 0.05$ in comparison of group 2E versus group 5E (Kruskal-Wallis test).

S4 TableE. Comparison of *G. duodenalis* and other intestinal pathogens positives: Alpha diversity index

Groups (n) Median [min-max] / Alpha diversity index	Group A (8) Control group	Group D (11) *Blastocystis and E. hartmanni* w/o pathogens	Group M (5) Bacteria andviruses w/o *G. duodenalis* w/o *Entamoeba* spp.
Chao1	150.70 [100.40−278.80]	271.90 [126.60−454.70]	154.20 [105.50−209.90]
Observed OTUs	125.00 [82.00−201.00]*	243.00 [122.00−336.00]	130.00 [94.00−170.00]
Faith's Phylogenetic Diversity	11.42 [8.457−15.23]*	21.39 [8.925−27.77]	13.63 [7.372−15.67]
Shannon	3.138 [1.514−3.817]*	4.254 [3.699−4.589] §	3.045 [2.315−3.308]

Only groups with a statistically significant difference were indicated.
Groups A: negative subjects; Groups Groups D: positive for *Blastocystis and En. hartmanni* without other pathogens; Groups M: positive for *Blastocystis and* mixed infection of pathogenic bacteria and viruses, without *G. duodenalis* and *Entamoeba* spp..

* means $p < 0.05$; A versus D; § means $p < 0.05$ D versus M. (Kruskal-Wallis test)
w/o means without.
