# Supplementary material for: Analysis of Human Gut Microbiota Composition Associated to the Presence of Commensal and Pathogen Microorganisms in Côte d’Ivoire
Source: Microorganisms. 2021 Aug 18;9(8):1763. doi: 10.3390/microorganisms9081763 (PMC8400437; doi:10.3390/microorganisms9081763)
Supplement: Supplementary file 1 [file microorganisms-09-01763-s001.zip › microorganisms-1330453-supplementary/Table S5A-E.pdf]

Table S5A. Beta diversity analysis for *Entamoeba* spp. presence.

Group	Family	Median [min-max]	p_value
negative	Bacteroidaceae	0.00425 [0-0.5432]	0.0444
En. coli,| *En. dispar*|, *En. hartmanni*		0.00125 [0-0.01]	
negative	Christensenellaceae	0.0015 [0-0.058]	0.0046
En. coli,| *En. dispar*|, *En. hartmanni*		0.01175 [0-0.0932]	
negative	Lachnospiraceae	0.1255 [0.0065-0.7335]	0.0390
En. coli,| *En. dispar*|, *En. hartmanni*		0.0855 [0.014-0.2075]	
negative	Rikenellaceae	0.00325 [0-0.0537]	0.0180
En. coli,| *En. dispar*|, *En. hartmanni*		0.01 [0-0.223]	
negative	Ruminococcaceae	0.2385 [0.001-0.8527]	0.0358
En. coli,| *En. dispar*|, *En. hartmanni*		0.405 [0.05-0.912]	

Family comparison in >1 year age subjects: negative (34 subjects) versus *En. coli*|*En. dispar*|*En.a hartmanni* positive (33 subjects). Only families producing a statistically significant difference ($p < 0.05$) were indicated.

Table S5B. Beta diversity analysis for *Entamoeba* spp. presence.

<table>
<tr><th>Group</th><th>Genus</th><th>Median [min-max]</th><th>p_value</th></tr>
<tr><td>negative</td><td rowspan="2">Bacteroides</td><td>0.042 [0-0. 5432]</td><td rowspan="2">0.044</td></tr>
<tr><td>En. coli,| En. dispar|, En. hartmanni</td><td>0.0012 [0-0.01]</td></tr>
<tr><td>negative</td><td rowspan="2">Christensenellaceae R-7 group</td><td>0.0005 [0-0.041]</td><td rowspan="2">0.0094</td></tr>
<tr><td>En. coli,| En. dispar|, En. hartmanni</td><td>0.0117 [0-0.0902]</td></tr>
<tr><td>negative</td><td rowspan="2">Rikenellaceae RC9 gut group</td><td>0 [0-0.0197]</td><td rowspan="2">0.0076</td></tr>
<tr><td>E. coli|E. dispar|E. hartmanni</td><td>0.0087 [0-0.2222]</td></tr>
<tr><td>negative</td><td rowspan="2">Ruminococcaceae UCG-002</td><td>0.0152 [0-0.685]</td><td rowspan="2">0.0009</td></tr>
<tr><td>E. coli|E. dispar|E. hartmann
E. coli|E. dispar|E. hartmanni i</td><td>0.154 [0-0.8272]</td></tr>
<tr><td>negative</td><td rowspan="2">Ruminococcaceae UCG-010</td><td>0.0002 [0-0.013]</td><td rowspan="2">0.0012</td></tr>
<tr><td>E. coli|E. dispar|E. hartmanni</td><td>0.008 [0-0.0997]</td></tr>
</table>

Genus comparison in >1 year age subjects: negative (34 subjects) versus *En. coli|En. dispar|En. hartmanni* positive (33 subjects). Only Genera producing a statistically significant difference ($p < 0.05$ or $p < 0.001$) were indicated.

Table S5C. Comparison of *E. hartmanni* groups: Beta diversity at genus level.

Groups (n) / Median [min-max] / Genus	Control group (8)	*E. hartmanni +Blastocystis* group (11)	*E. hartmanni+Blastocystis* +Pathogen group (9)
Alloprevotella	0 [0-0]**	0[0-0.11]	0 [0-0.01]
Bifidobacterium	0 [0-0.12]	0 [0-0.01]	00[0.01-0.36]§
Christensenellaceae R-7 group	0 [0-0.01]	0.01[0-0.07]°	0.01[0-0.06]
Faecalibacterium	0.18 [0.04-0.57]	0.06[0.02-0.28]°°	0.03[0.01-0.14]
Rikenellaceae RC9 gut group	0 [0-0]*	0.02[0-0.22]	0[0-0.04]
Ruminococcaceae UCG-002	0 [0.01-0.08]*	0.11[0-0.40]°°	0.21[0.01-0.43]
Succinivibrio	0 [0-0]*	0.02[0-0.08]	0[0-0.43]

Control group versus Group of subjects positive for *E. hartmanni* and *Blastocystis* . and versus Group of subjects positive for *E. hartmanni+Blastocystis* group versus *E. hartmanni+Blastocystis*; ° means $p < 0.05$ and °° means $p < 0.01$ Control group versus *E. hartmanni* and *Blastocystis* and Pathogen; § means $p < 0.05$ *E. hartmanni* and *Blastocystis* and other pathogens.

* means $p < 0.05$ and ** means $p < 0.01$ Control versus *E. hartmanni* and *Blastocystis* and Pathogen (Kruskal-Wallis test).

Table S5D. Beta diversity analysis for *Entamoeba* spp. detection

Group	Family	Median [min-max]	1E versus 3E	2E versus 3E
1E	Bacteroidaceae	0.0057 [0.002-0.223]	0.0312	0.0417
2E		0.0034 [0-0.5432]		
3E		0.0002 [0-0.004]		
1E	Bacteroidales S24-7 group	0 [0-0.0095]	0.0335	>0.05
2E		0.001 [0-0.1615]		
3E		0.01 [0-0.043]		

Family comparison in >1 year age subjects: 1E = Control group (8 negative subjects), 2E= not harboring *Entamoeba* species (20 subjects), 3E= carrying *En. coli* (10 subjects). Only comparisons producing a statistically significant difference ($p < 0.05$) were indicated. Only groups and Families producing a statistically significant difference were indicated.

Table S5E. Beta diversity analysis for *Entamoeba* spp. presence.

Genus (n)	1E Median [min-max]	2E Median [min-max]	3E Median [min-max]	5E Median [min-max]
[*Ruminococcus*] torques group	0.0031 [0-0.0517]	0.0029 [0-0.0038]*	0.0004 [0-0.0022]	0.0027 [0.001-0.0042]
Bacteroides	0.0057 [0.0002-0.223]*	0.0034 [0-0.5432]*	0.0002 [0-0.004]	0.0025 [0-0.01]
Lachnoclostridium	0.0025 [0.0007-0.0702]**	0.002 [0-0.1667]**	0 [0-0.0007]	0.0012 [0-0.006]
Ruminococcaceae UCG-002	0.0116 [0.0005-0.082]	0.15.61 [0-0.0151]§	0.1352 [0-0.8272]	0.214 [0.0007-0.4325]
Ruminococcaceae UCG-010	0 [0-0.001]§§	0.0038 [0-0.013]§	0.01 [0-0.0607]	0.0112 [0.0007-0.072]

Genus comparison in >1 year age subjects: Group 1E: control group (8 negative subjects); Group: 2E: 20 positive for pathogens not including *Entamoeba* spp.; Group 3E: 10 subjects positive for *En. coli* and other pathogens; Group 5E:9 subjects positive for *En. hartmanni* and other pathogens. Only groups and Genera producing a statistically significant difference were indicated.

*p value < 0.05; **p value < 0.01 by 1E and 2E versus 3E (comparison by Kruskal-Wallis test).

§p value < 0.05; §§p value < 0.01, 1E and 2E versus 5E (comparison by Kruskal-Wallis test).
